# Inhibitive effects of phytic acid combined with glutathione on the browning and oxidation of King Oyster mushroom (*Pleurotus eryngii*) slices during drying and storage

**DOI:** 10.1016/j.fochx.2023.100874

**Published:** 2023-09-12

**Authors:** Chao Gong, Wenjuan Gao, Shengjun Wu

**Affiliations:** a Jiangsu Key Laboratory of Marine Bioresources and Environment/Jiangsu Key Laboratory of Marine Biotechnology, Jiangsu Ocean University, Haizhou, China; b Co-Innovation Center of Jiangsu Marine Bio-industry Technology, Haizhou, China

**Keywords:** Browning, Oxidation, Drying, Storage

## Abstract

Phytic acid and glutathione can inhibit polyphenoloxidase (PPO) activity and suppress browning. This study investigated the effects of phytic acid alone (Treatment-1) or combined with glutathione (Treatment-2) on inhibiting browning and oxidation resistance of King Oyster mushroom **(***Pleurotus eryngii*) slices during drying and storage. In King Oyster mushroom slices, 0.08% phytic acid combined with 0.1% glutathione inhibited the PPO activity by 97.6%, suppressed browning by 78.09% after 6 h of drying at 60 °C and inhibited browning by 69.93% and oxidation by 78.75% after 12 months of storage at ∼ 20 °C. Results indicated that using phytic acid combined with glutathione may inhibit browning and suppress the oxidation of King Oyster mushroom slices during drying and storage.

## Highlights

• Glutathione and phytic acid effectively inhibited PPO activity.
• Glutathione and phytic acid inhibited browning of mushroom during drying.
• Glutathione and phytic acid inhibited browning of mushroom during storage.
• Glutathione and phytic acid decreased MDA of mushroom during storage.

## 1 Introduction

King Oyster mushroom (*Pleurotus eryngii*) is a large and rare edible fungus with thick meat, crisp texture and rich nutrition, which has been successfully cultivated and produced in recent years (Gao et al., 2022). This species is rich in carbohydrates, protein, vitamins, amino acids, sterols, calcium, magnesium, copper and zinc (Zhou, Li, Zhang, Hu, & Zou, 2022). King Oyster mushroom has many health functions, such as hypolipidemic activity (Zhao et al., 2023), gastrointestinal function (Ma et al., 2022) as well as antioxidant and anti‐fatigue activities (Zhao et al., 2020). As a healthy food in China, King Oyster mushroom is deeply loved by people, and it has a high development value (Liang, Huang, Mau, & Chiang, 2020).

Fresh King Oyster mushroom has a high water content and vigorous physiological metabolism, and it is prone to decay, browning and texture deterioration after harvest, resulting in a decline in quality (Lee et al., 2013). Therefore, the preservation and processing after picking has become an urgent problem for the development of the King Oyster mushroom industry (Lee et al., 2013). Drying is an important method for processing and storing of King Oyster mushroom. Slicing before drying destroys the original tissue of fresh King Oyster mushroom, resulting in enzymatic browning reaction of polyphenoloxidase (PPO) and phenolic substrates under aerobic conditions and oxidation of carbohydrates and fats, thereby accelerating the occurrence of King Oyster mushroom browning, affecting the quality of King Oyster mushroom slices and leading to flavour deterioration, nutrition reduction and loss of economic value (Su, Lv, Wang, Wang, & Li, 2020). Therefore, the colour after slicing and before drying must be protected to prevent browning reaction.

Phytic acid and glutathione are antioxidants that can inhibit PPO activity and browning (Wu, 2014, Fang et al., 2022). Phytic acid has been applied to suppress the browning of fresh-cut apples (Fang et al., 2022), chestnut (Li et al., 2017) and apple juice (Du, Dou, & Wu, 2012). Glutathione has been used to inhibit the browning of apple (Billaud, Brun-Mérimée, Louarme, & Nicolas, 2004) and grape juice (Wu, 2014). In view of these data, phytic acid combined with glutathione can suppress the browning of King Oyster mushroom slices during drying and storage. Thus, this study aimed to explore the suppressive effects of combining phytic acid with glutathione on the browning of King Oyster mushroom slices during drying and storage.

## 2 Materials and methods

### 2.1 Materials

Fresh King Oyster mushrooms were purchased from a farm product market and stored at 5 °C until use. Phytate acid with a purity of > 99% and glutathione with a purity of > 99% were purchased from China Pharmaceutical Group Co., Ltd. (Beijing, China).

### 2.2 Preparation of PPO extract from King Oyster mushroom

One hundred grams of King Oyster mushroom was added to 200 mL of glacial acetone (approximately − 20 ℃) and homogenised using a homogeniser for 5 min. The mixture was filtered using a medium-speed filter paper, and the residue was washed repeatedly with glacial acetone and filtered until it became a white powder, which was acetone powder. One gram of acetone powder was added to 20 mL of 0.025 mol/L phosphate-buffered solution (pH7.2), homogenised using a magnetic stirrer at 0 ℃ for 30 min and centrifuged at 12,000 × *g* for 30 min. Afterwards, the supernatant was collected to yield crude enzyme extract. One gram of acetone powder was added to 20 mL of 0.025 mol/L phosphate-buffered solution (pH 7.2), homogenised using a magnetic stirrer at 0 ℃ for 30 min and centrifuged at 12,000 × *g* for 30 min. Then, the supernatant was taken as a crude enzyme extract. NH_4_SO_4_ was added into the crude enzyme extract to 80% saturated solution and stirred for 30 min. Next, the mixture was placed overnight, centrifuged at 12,000 × *g* for 30 min, precipitated and dissolved in 0.025 mol/L phosphate buffer (pH 7.2) and dialysed with 10 mmol/L Tris-HCl solution (pH 8.3) for 18 h. During dialysis, Tris-HCl solution was replaced three times, and the final product was purified PPO.

### 2.3 Determination of PPO activity

2.75 mL of 0.025 mol/L phosphate-buffered solution (pH 7.2) was added into a 25 px colorimetric cup containing 0.1 mL of 0.15 mol/L catechol solution, mixed well and placed at room temperature for 3 min. Then, 0.1 mL of enzymatic solution was added. After 5 s, the change of the A420 value within 1 min was recorded. The amount of enzyme required to change the light absorption value by 0.001 per minute was one activity unit.

### 2.4 Suppressive effects of phytic acid combined with glutathione on PPO activity

Different concentrations of phytic acid solution (0% to 0.12%) were used to assess PPO activity. One hundred microliters of crude PPO extract was mixed with 100 µL of phytic acid solution. After the mixture was incubated at ∼ 25 °C for 30 min, 400 µL of 0.05 mol/L of assay phosphate buffer (pH 6.0) and 600 µL of 15 mmol/L l-DOPA (45 °C) were added to initiate the reaction, which was performed at 45 °C. Absorbance was recorded at 475 nm for 3 min. The control experiments were performed using the same method; however, the modulating agent used was replaced with deionised. l-DOPA was replaced with distilled water to prepare the blank sample. An increase in the absorbance by 0.001 at 475 nm/min/mL was used to define a unit of PPO activity (1 U). The inhibitory activity was calculated in accordance with the following equation (1):

(1) Inhibition (%) = 100 × (A − B)/A,

where A is the initial enzymatic activity in the control, and B is the residual enzymatic activity in the presence of an inhibitor.

The maximum inhibition level of phytic acid (0.08%) combined with different concentrations of glutathione solution (0% to 0.12%) was evaluated for PPO inhibitory activity.

### 2.5 Treatment of King Oyster mushroom slices

Fresh King Oyster mushrooms of the same size were washed and cut into slices with a similar thickness of 5 mm. Purified water (control), 0.08% phytic acid (Treatment-1) and 0.08% phytic acid + 0.1% glutathione (Treatment-2) were used as dipping solutions. A total of 400 King Oyster mushroom slices were randomly and evenly assigned to the control and treatment groups. The King Oyster mushroom slices were soaked in the two dipping solutions at 5 °C for 20 min. After draining the residual dipping solutions, the King Oyster mushroom slices were dried in an air-drying oven at 60 °C for 6 h, during which the colour of King Oyster mushrooms was measured every 1 h. The dried King Oyster mushroom slices were cooled and stored at ∼ 25 °C for 10 months, during which the colour and malondialdehyde (MDA) content of King Oyster mushrooms were measured every 2 months.

### 2.6 Colour determination

The colour of the King Oyster Mushroom slices was measured by using a colorimeter (CS-100A, Minolta Co., Ltd., Japan). The colour parameters were L* (brightness), a* (red/green) and b* (yellow/blue). The total colour difference (Δ*E*) of the King Oyster Mushroom slices was calculated in accordance with Equation (2).

(2) $$\Delta E=\sqrt{{\left(\Delta L^{*}\right)}^{2}+{\left(\Delta a^{*}\right)}^{2}+{\left(\Delta b^{*}\right)}^{2}}$$

where ΔL*, Δa* and Δb* indicate the differences amongst the lightness, red/green and yellow/blue of the sample and standard, respectively.

### 2.7 MDA determination

MDA condensed with thiobarbituric acid to form a red product with a maximum absorption peak at 532 nm. The content of lipid peroxide in the sample can be estimated by colorimetry. The absorbance at 600 nm was measured. The content of MDA was calculated by the difference between the absorbance at 532 and 600 nm.

### 2.8 Statistical analysis

All experiments were repeated six times. The data were expressed as mean ± error (SE). All data with the same drying or storage time were statistically analysed by using one-way analysis of variance and Tukey Kramer multi-range test.

## 3 Results and discussion

### 3.1 Effect of glutathione alone or combined with phytic acid on the inhibition of PPO activity

**Fig. 1:**
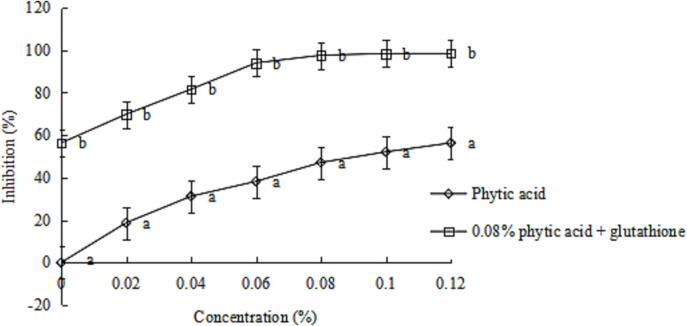
Effect of phytic acid alone or combined with glutathione on the inhibition of PPO activity of *P. eryngii*. Data are shown as mean ± SD (n = 6). The different letters on the bars indicate significant differences (*P* < 0.05).

The PPO activity can be inhibited by varying agents such as a reducing agent, chelating agent, complexing agent, acidulants, enzymatic inhibitor and enzymatic treatment agent (Zhou et al., 2016). The inhibition effects of phytic acid alone or in combination with glutathione on PPO activity in King Oyster mushroom are presented in Fig. 1. Different levels of phytic acid exhibited the inhibition effect on PPO activity, and the maximal inhibition rate (56.31%) was obtained at 0.08%. The inhibition rate increased from 56.31% to 97.62% as glutathione solutions at different concentrations ranging from 0% to 0.01% were added. However, the inhibition rate did not further increase if the concentration of phytic acid exceeded 0.08%. Thus, 0.08% phytic acid combined with 0.1% glutathione exhibit a maximum inhibition rate of PPO.

### 3.2 Effect of phytic acid alone or in combination with glutathione on the browning of the King Oyster mushroom slices during drying

**Fig. 2:**
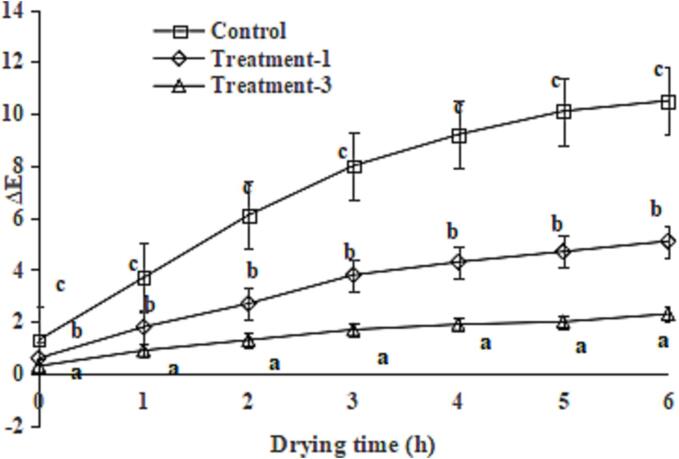
Effect of glutathione combined with phytic acid on browning degree of *P. eryngii* slices during drying. Data are shown as mean ± SD (n = 6). The different letters on the bars indicate significant differences (*P* < 0.05).

The changes in Δ*E* values of the King Oyster mushroom slices during drying are presented in Fig. 2. The Δ*E* values of the King Oyster mushroom slices in the control group increased sharply with drying time (*P* < 0.05). In addition, the Δ*E* values of the King Oyster mushroom slices in the treatment groups increased slowly, and they were lower than those of the control group after 6 h of drying, indicating that phytic acid alone or phytic acid combined with glutathione effectively suppressed the enzymatic browning of King Oyster mushroom slices during drying. The differences in the Δ*E* values between treatment-1 and treatment-2 groups increased during 6 h of dry; this could be due to the PPO activity and browning inhibition ability of glutathione (Wu, 2014) and showed a synergistic effect of phytic acid and glutathione. Similarly, phytic acid suppressed the enzymatic browning of fresh-cut apples (Fang et al., 2022) and chestnut (Li et al., 2017), whilst glutathione inhibited the enzymatic browning of *Agaricus bisporus* slices during hot-air drying (Xia, 2013).

### 3.3 Effect of phytic acid alone or in combination with glutathione on the browning of the King Oyster mushroom slices during storage

**Fig. 3:**
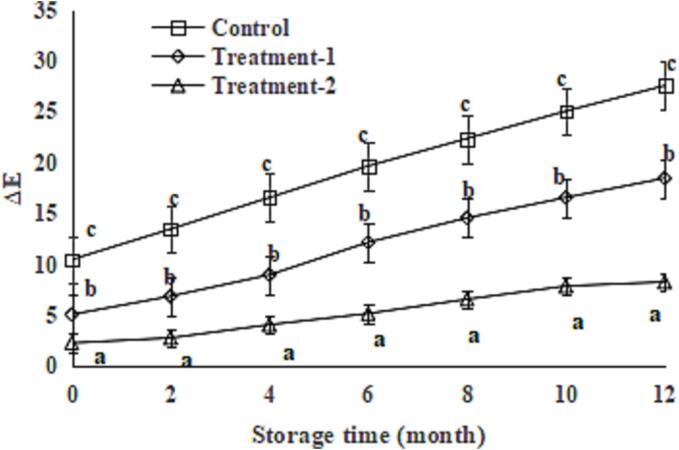
Effect of glutathione combined with phytic acid on browning of *P. eryngii* slices during storage. Data are shown as mean ± SD (n = 6). The different letters on the bars indicate significant differences (*P* < 0.05).

Given the low drying temperature, some PPO activities are still observed in the King oyster mushroom slices. Therefore, enzymatic browning still occurred in mushroom slices during storage. In addition, non-enzymatic browning occurred in the mushroom slices during storage. Non-enzyme browning includes Maillard reaction, oxidative decomposition of ascorbic acid, oxidative condensation of polyphenols, caramelisation reaction, discoloration of five-colour anthocyanins and browning caused by metal ions. The Δ*E* values of the King Oyster mushroom slices in the control group increased steadily from 10.51 to 27.62 during storage (Fig. 3, *P* < 0.05). However, the Δ*E* values the King Oyster mushroom slices in the treatment groups increased slowly (from 5.12 to 18.51 for treatment-1 and from 2.28 to 8.29 for treatment-2), and they were lower than those of the control group after 12 months of storage (Fig. 3, *P* < 0.05), indicating that phytic acid combined with glutathione effectively suppressed the enzymatic browning and non-enzymatic browning of King Oyster mushroom slices during drying. The differences in the Δ*E* values between treatment-1 and treatment-2 groups increased during 12 months of storage; this also could be due to the PPO activity and browning inhibition ability of glutathione (Wu, 2014) and showed a synergistic effect of phytic acid and glutathione. Similarly, phytic acid suppressed the enzymatic and non-enzymatic browning of apple juice (Du et al., 2012), whilst glutathione inhibited the enzymatic and non-enzymatic browning of grape juice (Wu, 2014).

### 3.4 Effect of phytic acid alone or in combination with glutathione on MDA content in the King Oyster mushroom slices during storage

**Fig. 4:**
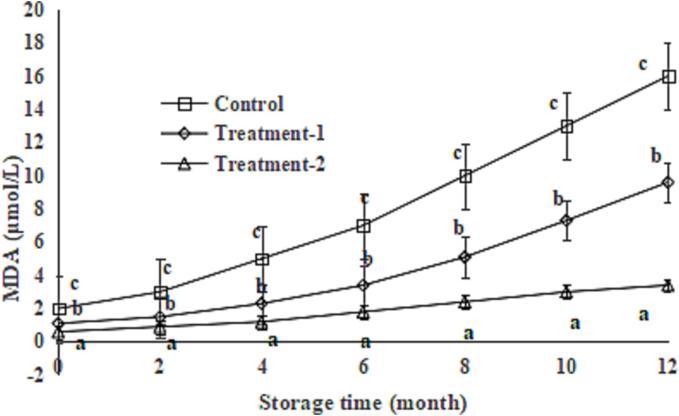
Effect of glutathione combined with phytic acid on TBA level of *P. eryngii* slices during storage. Data are shown as mean ± SD (n = 6). The different letters on the bars indicate significant differences (*P* < 0.05).

Dried King Oyster mushroom contains a small amount of easily oxidised lipids (Zhang, Zhang, Liu, Zhang, & Sun, 2013). The oxidation of lipids will lead to the deterioration of King Oyster mushroom slices*.* Therefore, lipid should be prevented from oxidation during storage. The MDA content in the King Oyster mushroom slices in the control group increased sharply from 2.14 µmol/L to 16.09 µmol/L during storage (Fig. 4, *P* < 0.05). Nevertheless, the MDA contents in the King Oyster mushroom slices in the treatment groups increased slowly (from 1.07 µmol/L to 9.58 µmol/L for treatment-1 and from 0.61 µmol/L to 3.08 for treatment-2 µmol/L), and they were lower than that of the control group after 12 months of storage (Fig. 4, *P* < 0.05), indicating that phytic acid alone or combined with glutathione effectively inhibited the lipid oxidation of King Oyster mushroom slices during drying. The differences in the MDS contents between treatment-1 and treatment-2 groups increased during 12 months of storage; this could be due to the antioxidant activity of glutathione (Wu, 2014) and showed a synergistic effect of phytic acid and glutathione.

## 4 Conclusions

Browning and oxidation decrease the commercial value of King Oyster mushrooms. In this study, 0.08% sodium phytic acid combined with 0.1% glutathione suppressed browning during drying and storage and inhibited the lipid oxidation of King Oyster mushrooms during storage. Thus, treatment with 0.08% sodium phytic acid combined with 0.1% glutathione may be a practical method to prepare and store dried King Oyster mushroom slices.

## CRediT authorship contribution statement

**Chao Gong:** Investigation, Data curation, Formal analysis, Methodology, Software, Writing – original draft. **Wenjuan Gao:** Investigation, Methodology, Software, Validation, Visualization. **Shengjun Wu:** Conceptualization, Resources, Supervision, Funding acquisition, Investigation, Project administration, Writing – original draft, Writing – review & editing.

## Declaration of Competing Interest

The authors declare that they have no known competing financial interests or personal relationships that could have appeared to influence the work reported in this paper.

## Data Availability

The authors do not have permission to share data.
